# Antimicrobial Activity and Action Mechanism of Thymoquinone against *Bacillus cereus* and Its Spores

**DOI:** 10.3390/foods10123048

**Published:** 2021-12-08

**Authors:** Shuo Wang, Haichao Deng, Yihong Wang, Wushuang Rui, Pengyu Zhao, Qiyao Yong, Du Guo, Jie Liu, Xinyi Guo, Yutang Wang, Chao Shi

**Affiliations:** College of Food Science and Engineering, Northwest A&F University, Xianyang 712100, China; wangshuoddu@126.com (S.W.); haichaodeng99@163.com (H.D.); 2018013432@nwafu.edu.cn (Y.W.); rivulet@nwafu.edu.cn (W.R.); zhaopengyu1999@163.com (P.Z.); yqy@nwafu.edu.cn (Q.Y.); duguo2911@163.com (D.G.); liujie2019013313@nwafu.edu.cn (J.L.); guoxinyi@nwafu.edu.cn (X.G.); wyt991023@nwsuaf.edu.cn (Y.W.)

**Keywords:** thymoquinone, *Bacillus cereus*, antimicrobial activity, reconstituted infant formula, toxin-related gene, spores

## Abstract

In this study, thymoquinone (TQ), a natural active substance, was investigated for its antibacterial activity against *Bacillus cereus*, and its inhibitory effect on *B. cereus* in reconstituted infant formula (RIF) was evaluated. In addition, the inhibitory effect of TQ on *B. cereus* spore germination was explored. The minimum inhibitory concentrations (MICs) and minimum bactericidal concentrations (MBCs) of TQ against eight *B. cereus* strains ranged from 4.0 to 8.0 μg/mL, whereas *B. cereus* treated with TQ displayed a longer lag phase than the untreated control. TQ exerted a good bactericidal effect on *B. cereus* in Luria–Bertani broth. In addition, TQ obviously reduced the intracellular ATP concentration of *B. cereus*, which caused depolarization of the cell membrane, increased the intracellular reactive oxygen species level, impaired the cell morphology, and destroyed proteins or inhibited proteins synthesis. This provides a mechanism for its bacteriostatic effect. TQ also inactivated *B. cereus* growth in RIF. Moreover, reverse transcription–quantitative polymerase chain reaction illustrated that TQ downregulated the transcription of genes related to hemolysin, non-hemolytic enterotoxin, enterotoxin, and cytotoxin K. Meanwhile, TQ displayed the ability to inhibit the germination of *B. cereus* spores. These findings indicate that TQ, as an effective natural antimicrobial preservative, has potential applications in controlling food contamination and foodborne diseases caused by *B. cereus*.

## 1. Introduction

*Bacillus cereus* is a Gram-positive, flagellated, aerobic endospore-forming foodborne pathogenic bacterium that is non-susceptible to extreme environmental conditions, including heat, freezing, drying, and radiation [1]. *B. cereus* strains that can cause poisoning usually pollute cereals and recombinant infant formula (RIF), and they are also present in vegetables and meat [2,3,4]. Usually, contaminated food is not obviously rotten, which makes *B. cereus* contamination difficult to observe. Therefore, foodborne diseases caused by *B. cereus* are a matter of great importance for the food industry and consumers. *B. cereus* usually causes vomiting, diarrhea, and abdominal pain [5], although these gastrointestinal symptoms are mild and self-limiting. *B. cereus* can also cause diseases such as severe eye infections, osteomyelitis, hepatitis, and even death in severe cases [6]. In Belgium, *B. cereus* was the most frequently reported foodborne outbreak pathogen in 2018, and *B. cereus* was the second most common pathogen in foodborne outbreaks between 2007 and 2014 in France [7].

Spores are round or oval dormancy bodies with strong stress resistance that are formed by *Bacillus* bacteria when the external environment does not permit growth [8]. *B. cereus* spores can withstand high temperature, high pressure, and ultraviolet radiation; they have a certain level of resistance to toxic chemicals; and neither conventional cooking nor pasteurization procedures can effectively inactivate them [9]. Spores can easily germinate in response to nutrients, cationic surfactants, exogenous dipicolinic acid, and other factors [10]. Therefore, the germination of spores present in food may lead to the spoilage of pasteurized dairy products, meat, and vegetables, which poses great risks to consumers and the food industry. Although high-temperature thermal sterilization techniques such as distillation can effectively inactivate *B. cereus* and its spores in foods, excessive heating can impair the sensory quality and nutrient content of foods [11]. The improper use of chemical reagents can lead to harm in humans. Therefore, there is an urgent need to identify a safe and effective method for controlling contamination by *B. cereus* and its spores.

**Figure 1:**
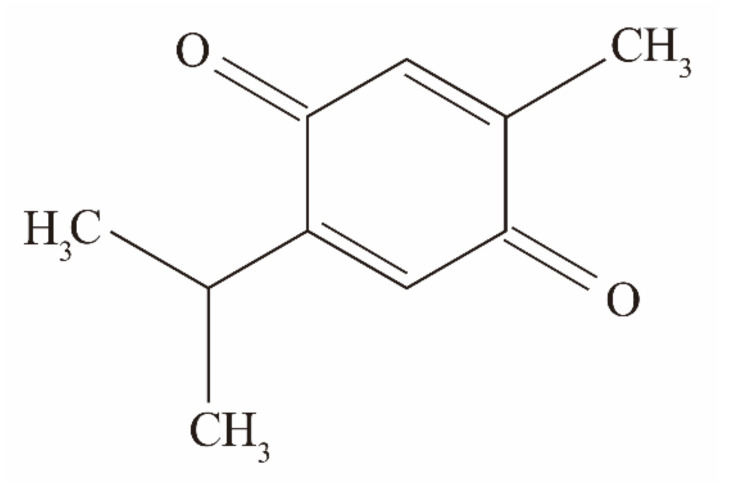
Chemical structure of thymoquinone.

With the frequent occurrence of food safety problems and the transformation of people’s consumption concepts, natural extracts have attracted increasing attention because of their natural nature, high biodegradability, and good specificity. Many phytochemicals are considered to have inhibitory effects on foodborne pathogens [12]. *Nigella sativa*, which possesses various biological activities, is often used to treat diseases such as hypertension, asthma, and skin disorders [13]. Despite being approved for use in food by the United States Food and Drug Administration, it is most commonly used as a spice in food production [14]. Thymoquinone (TQ,5-isopropyl-2-methyl-1,4-benzoquinone, C_10_H_12_O_2_; Figure 1) is an effective monomer isolated from *N. sativa*. Studies have identified a wide range of biological activities for TQ, such as antioxidant, antiatherosclerosis, and antitumor effects [15]. TQ has been reported to have strong activity against the bacteria *Cronobacter sakazakii* [16] and *Staphylococcus aureus* [17], and the ability of TQ to inhibit biofilm formation was demonstrated in 11 pathogenic bacteria including *Listeria monocytogenes* and *Escherichia coli* [18]. However, the antimicrobial activity of TQ against *Bacillus* and its spores has rarely been reported, and the antibacterial mechanism is unclear.

The antimicrobial and sporicidal effects of TQ against *B. cereus* were determined in this study. For this purpose, the minimum inhibitory concentration (MIC) and minimum bactericidal concentration (MBC) were first determined to evaluate the bacteriostatic effects of TQ. Subsequently, the growth curve, time–kill curve, membrane potential, intracellular ATP concentration, ROS level, cell morphology, and protein changes were analyzed to explore the possible inhibitory mechanism of TQ in vegetative cells. Moreover, the effect of TQ on the expression of toxin-related genes in *B. cereus* was investigated, and its effect on toxin synthesis was evaluated. Additionally, the inhibitory effect of TQ on *B. cereus* in contaminated infant formula was verified, and the industrial practicability of TQ was confirmed. Finally, the effect of TQ on spore germination was investigated to evaluate its inhibitory effect on spores.

## 2. Materials and Methods

### 2.1. Reagents

TQ (HPLC ≥ 98%, CAS: 490-91-5) was purchased from Tokyo Chemical Industry Co. (Tokyo, Japan). TQ was dissolved in dimethyl sulfoxide (DMSO). The final concentration of DMSO in all experiments was 0.10% (v/v). All other chemicals used in this study were of analytical grade.

### 2.2. Bacterial Strain, Growth Conditions, and Spore Preparation

*B. cereus* ATCC 14579 was obtained from the American Type Tissue Collection (Manassas, VA, USA). *B. cereus* CMCC 63301 and *B. cereus* CMCC 63303 were obtained from the National Center for Medical Culture Collection (Beijing, China). The strains *B. cereus* BR1 and *B. cereus* BR4 were isolated from rice purchased in a retail market and identified by members of our laboratory, using 16S rDNA sequencing. Three other strains of *B. cereus* (C7, C58, and M98) were obtained from Peng Fei, Henan University of Science and Technology and isolated from milk powder. All eight *B. cereus* strains were used in the MIC and MBC assays, while *B. cereus* ATCC 14579 was used in all the subsequent experiments because it contains the phenotypic and genotypic characteristics tested in subsequent experiments.

*B. cereus* stored at −80 °C in 15% glycerol and 85% Luria–Bertani (LB, Land Bridge Technology Co., Beijing, China) broth was streaked onto LB agar plates, followed by incubation at 37 °C for 24 h, and then one colony was inoculated into 30 mL of LB broth at 37 °C for 12 h with shaking at 130 rpm. The bacterial suspension was centrifuged (8000× *g*, 5 min, 4 °C), washed twice with sterile phosphate-buffered saline (PBS, pH 7.2), and resuspended in LB broth. The optical density at 600 nm (OD_600 nm_) of the bacterial suspension was measured by a spectrophotometer (SmartSpec Plus; Bio-Rad, Hercules, CA, USA). When the OD_600 nm_ value was 0.5, corresponding to a cell concentration of approximately 6 × 10^7^ colony-forming units (CFU)/mL.

Spore suspensions were prepared as described previously with slight modifications [19]. The bacterial suspension (100 μL) was formed as described in the previous paragraph in 30 mL LB broth at 37 °C for 12 h with shaking at 130 rpm, then was spread onto a magnesium nutrient agar medium (Qingdao Hope Bio-Technology Co., Ltd., Qingdao, China) and cultured at 37 °C for 7 days. An examination was performed under an oil microscope via phase microscopy to determine the formation of spores after staining. When the spore purity reached 90% or higher, a cotton swab was used to harvest the spore lawn from the medium, and the spores were resuspended in PBS. The suspension was centrifuged (8000× *g*, 5 min, 4 °C), washed 3 times with PBS, and heated at 80 °C for 10 min to kill the vegetative cells. To remove the vegetative cells, the spores were washed 3 times with PBS and then suspended in 10 mL of PBS to create a spore suspension. The spore concentration was determined by the viable count method, and the final spore suspension was stored at −20 °C.

### 2.3. Determinations of the MIC and MBC of TQ against B. cereus

To investigate the inhibitory effect of TQ on *B. cereus*, the MICs and MBCs of TQ against 8 strains of *B. cereus* were determined. The broth microdilution method advocated by the Clinical and Laboratory Standards Institute [20] was used to determine the MIC. Briefly, the prepared *B. cereus* suspension was diluted to 5 × 10^5^ CFU/mL with LB broth. Equivalent volumes (100 μL) of the bacterial suspension and serial dilutions of TQ in LB broth were added to a sterile 96-well microtiter plate. The final concentrations of TQ were 0 (control), 1, 2, 4, 8, and 16 μg/mL. OD_600 nm_ was measured using a microtiter plate reader (Model 680; Bio-Rad, Hercules, CA, USA), and the measurement was repeated after culturing the bacteria at 37 °C for 24 h. The MIC was defined as the lowest antimicrobial concentration of TQ corresponding to an OD_600_ change of <0.05. Next, 100-μL suspensions at concentrations equal to or greater than the MIC were spread on LB agar plates and cultured at 37 °C for 48 h. MBC was defined as the minimum concentration at which no visible colonies were observed in the LB agar plates. The tests of each strain were performed in triplicate independently.

### 2.4. Mechanism of Action against B. cereus

#### 2.4.1. Growth Curve

Growth curves were determined as described by Shi et al. [21], with some modifications. The prepared bacterial suspension was diluted to 5 × 10^6^ CFU/mL with LB broth and then mixed with the same volume (125 μL) of TQ solution to achieved final TQ concentrations of 0 (control), 1/32 MIC, 1/16 MIC, 1/8 MIC, 1/4 MIC, 1/2 MIC, MIC, 2 MIC, and 4 MIC. The mixture was further cultured at 37 °C, and bacterial growth was monitored at 1-h intervals with the OD_600_ determined using an automatic Bioscreen C instrument (Labsystems, Helsinki, Finland). Three independent replicates of each experiment were performed.

#### 2.4.2. Inactivation of *B. cereus* by TQ in LB Broth

Determination of the time–kill curves was based on the method described by Wang et al. [22], with slight modifications. TQ was mixed with the bacterial suspension at the adjusted concentration (approximately 1 × 10^6^ CFU/mL) to maintain the final concentrations of 0 (control), 1/2 MIC, MIC, 2 MIC, and 4 MIC. Samples were collected at 0, 1, 2, 4, 6, and 9 h after inoculation; continuously diluted in PBS; inoculated onto LB agar plates; and cultured at 37 °C for 12 h. After incubation, the *B. cereus* colonies were counted. Three independent replicates of each experiment were performed.

#### 2.4.3. Membrane Potential

The detection of the membrane potential of *B. cereus* ATCC 14579 was performed as described in Guo et al. [23]. In brief, *B. cereus* was resuspended in PBS to a concentration of approximately 6 × 10^7^ CFU/mL. Next, 125 μL of the bacterial suspension was transferred to a black 96-well microtiter plate and cultured at 37 °C for 30 min, then 1 μL of bis-(1,3-dibutyl barbituric acid) trimethine oxonol (DiBAC4(3); Molecular Probes, Eugene, OR, USA) was added to each well, and the black 96-well microtiter plate was incubated in the dark at 37 °C for 30 min. After incubation, the TQ solution was added at final concentrations of 0 (control), MIC, and 2 MIC. The fluorescence of each well was measured at excitation and emission wavelengths of 492 and 515 nm, respectively, after 5 min using a multifunctional microplate reader (Spectra Max M2; Molecular Devices, San Jose, CA, USA). The results were corrected according to the measured background fluorescence, and the membrane potential was expressed as relative fluorescence units (RFU, RFU = FU_treatment_ − FU_control_). Three independent replicates of each experiment were performed.

#### 2.4.4. Determination of Intracellular ATP Concentrations

The intracellular ATP concentration was determined according to the method described by Yang et al. [24], with slight modifications. After centrifugation, *B. cereus* ATCC 14579 was resuspended in PBS (approximately 1 × 10^8^ CFU/mL). One hundred milliliters of bacterial suspensions with different concentrations of TQ (0, MIC, 2 MIC) was cultured at 37 °C for 30 min. Next, each sample was placed on ice for ultrasonication, and the supernatant was collected by high-speed centrifugation. The supernatants were transferred instantly and stored on ice until use. The intracellular ATP concentration of each sample was obtained by determining the ATP content of supernatant using an ATP assay kit (Beyotime Bioengineering Institute, Shanghai, China). ATP standard solutions or the sample supernatant was mixed with an equal volume (100 μL) of ATP detection reagent in a white 96-well plate, and the light emission (bioluminescence) was measured using a multifunctional microplate reader. The standard curve and measured luminescence value were used to calculate the intracellular ATP concentrations. Three independent replicates were used for each experiment.

#### 2.4.5. ROS Determination

Intracellular ROS levels were determined as described by Jeon and Ha [25], with some modifications. Briefly, *B. cereus* was resuspended in PBS to a concentration of approximately 6 × 10^7^ CFU/mL. Different concentrations of TQ were then added to the bacterial suspension to achieve final concentrations of 0 (control), 1/4 MIC, 1/2 MIC, and MIC, and samples were incubated for 1 or 2 h. TQ was removed followed by the addition of the fluorescent probe CM-H_2_DCFDA, and after 15 min of incubation in a 37 °C incubator, the samples were washed twice with PBS (8000× *g*, 5 min, 4 °C). Fluorescence was measured spectrophotometrically at excitation and emission wavelengths of 495 and 520 nm, respectively, and individual samples were subjected to plate counting to obtain the relative fluorescence values. Three independent replicates of each experiment were performed.

#### 2.4.6. Bacterial Morphology

Observation of the morphology of *B. cereus* treated with TQ was performed by field emission scanning electron microscopy (FESEM) using a previously reported method [26], with some modifications. Overnight cultures treated with TQ (0, 2 MIC, 4 MIC) were incubated at 37 °C for 2 or 4 h, harvested by centrifugation at 8000× *g*, and washed twice with PBS. These bacteria were resuspended in PBS containing 2.5% (v/v) glutaraldehyde and incubated at 4 °C for 12 h for immobilization. After washing with PBS and sterile water, the microorganisms were gradient-dehydrated using water–alcohol solutions with different concentrations (30%, 50%, 70%, 80%, 90%, and 100% ethanol) and shaken for 10 min. The cells were dropped onto small glass slides and dried overnight. These samples were fixed on a support for drying and vacuum gold spraying, and finally observed using a field emission scanning electron microscope (S-4800; Hitachi, Tokyo, Japan) at ×15,000 magnification.

#### 2.4.7. Sodium Dodecyl Sulfate–Polyacrylamide Gel Electrophoresis (SDS-PAGE)

The intracellular soluble proteins of *B. cereus* ATCC 14579 were visualized using SDS-PAGE according to the method established by Kang and Song [27], with slight modifications. Bacterial suspensions exposed to TQ (0, MIC, 2 MIC, 4 MIC) were cultured at 37 °C for 12 h. Next, cell pellets were obtained via centrifugation at 10,000× *g* for 5 min and rinsed with PBS. Protein extraction was performed using a Gram-positive bacterial protein extraction kit (BestBio Biotechnology Co., Ltd., Shanghai, China). The supernatant was retained, and the protein concentration was determined using a Bicinchoninic Acid Protein Assay Kit (Kangwei Century Biotechnology Co., Ltd., Jiangsu, China). Next, 20 μL of a 5× loading buffer (containing 100 mM Tris-HCl, pH 6.8, 10% (*w/v*) SDS, 50% (*w/v*) glycerol, 200 mM dithiothreitol, 0.5% (*w/v*) bromophenol blue) was added to 80 μL of the sample supernatant, which had been adjusted to a consistent protein concentration. The samples were then boiled for 5 min, and 10 μL of each sample was used for SDS-PAGE after cooling to room temperature. The intracellular soluble proteins of *B. cereus* were separated by electrophoresis with a 5% (*v/v*) stacking gel and a 12.5% (*v/v*) separating gel, followed by staining with Coomassie brilliant blue R-250. After decolorization, gel imaging electrophoresis was used for observation.

#### 2.4.8. Reverse Transcription–Quantitative PCR (RT-qPCR)

The transcription levels of nine genes (Table 1) were detected by RT-qPCR to confirm the effect of TQ on the toxin expression of *B. cereus* at the gene level. *B. cereus* ATCC 14579 was exposed to TQ (0, 1/8 MIC, 1/4 MIC) at 37 °C for 12 h. The cultures were centrifuged at 8000× *g* for 5 min, the supernatant was removed, and the precipitate was suspended in PBS. Bacterial total RNA was collected according to the instructions of the RNA Prep Pure Bacteria Kit (Tiangen, Beijing, China). The RNA concentrations were determined using a nucleic acid and protein spectrophotometer (Nano-200; Aosheng Instrument Co., Ltd., Hangzhou, China). The RNA of the samples was reverse-transcribed into cDNA using the PrimeScript RT Master Mix reagent kit (TaKaRa, Beijing, China), following the manufacturer’s instructions. The primer sequences for RT-PCR of *B. cereus* re listed in Table 1. Stably expressed 16S rRNA was used as a housekeeping gene. The reactions in a 25-μL system were performed using SYBR Premix Ex Taq™ II (TakaRa, Beijing, China). The reaction was started at the initial denaturation temperature of 95 °C for 30 s, followed by 40 amplification cycles of 95 °C for 30 s and 60 °C for 30 s, and dissociation at 95 °C for 15 s and 60 °C for 30 s. The cycle threshold (Ct) was measured for the relative gene expression and analyzed using the 2^−∆∆Ct^ method. Three independent replicates of each experiment were performed.

### 2.5. Determination of the Antibacterial Activity of TQ in Reconstituted Infant Formula (RIF)

RIF (Feihe, Qiqihaer, China) was purchased from a local market (Yangling, China). RIF was prepared according to the manufacturer’s instructions, and 15 g of powder was dissolved in 100 mL of sterile water and pasteurized at 63 °C for 30 min to eliminate background microbial communities [22]. The prepared suspensions of *B. cereus* ATCC 14579 were added to RIF to reach a concentration of approximately 7.0 log CFU/mL. TQ solutions were added to the samples at final concentrations of 0 (control), 20, 40, and 80 μg/mL. Subsequently, the prepared samples were incubated at 25 °C for 0, 1, 2, 3, 4, 6, or 9 h, and the viable counts were determined using the LB plate counting method. Three independent replicates of each experiment were performed.

### 2.6. Inhibitory Effect of TQ on B. cereus Spores

#### 2.6.1. Effect of TQ on Spore Germination Rates

The effect of TQ on the germination rate of *B. cereus* spores was determined as described by Shu et al. [28]. Equal volumes of the spore suspension (5 × 10^6^ CFU/mL) and PBS with different concentrations of TQ were mixed to achieve final concentrations of 0 (control), MIC, 2 MIC, and 4 MIC. The mixture was incubated at 37 °C with shaking (130 rpm) for 2 h. The suspensions were centrifuged (8000× *g*, 4 °C, 5 min) and washed twice with PBS. The cultures were then resuspended in PBS, and a 10-fold serial dilution was used to obtain the solutions for counting. Next, 100 μL of the diluent was coated onto an LB agar plate and cultured at 37 °C for 24 h. The spore germination rate ((treatment group/control group) ×100%) was obtained by counting the colonies on the plates, which indicated the sporicidal activity of TQ. Three independent replicates of each experiment were performed.

#### 2.6.2. Confocal Laser-Scanning Microscopy Observations

The nucleic acid dye SYTO 16 was used to observe the effects of TQ on the spore germination of *B. cereus*, as described by Kong et al. [29], with minor modifications. The spores were washed with 0.85% NaCl solution and sterile water, centrifuged (8000× *g*, 4 °C, 5 min), and then resuspended with sterile water. Samples were incubated in a dry bath at 65 °C for 20 min and then placed on ice for 15 min to activate spores. After activation, the spores were transferred to a 25 mM Tris-HCl buffer (pH 7.4) containing 1 mM l-alanine for germination. At the same time, TQ was added to the spores at final concentrations of 0 (control), MIC, 2 MIC, and 4 MIC. Finally, 3 μL of SYTO 16 was added per milliliter of the cell sample, and the samples were fully mixed in the dark at room temperature for 30 min. A drop of the cell suspension was placed on the slide and observed via confocal laser-scanning microscopy (CLSM) (A1; Nikon, Tokyo, Japan).

### 2.7. Statistical Analysis

All experiments were independently performed in triplicate. All data are presented as the mean ± standard deviation. Differences between means were tested using Student’s *t*-test and analyzed using IBM SPSS software (version 19.0; SPSS Inc., Chicago, IL, USA). Significance was indicated by *p* < 0.05, and *p* < 0.01 was considered to indicate extreme significance. Bars in graphs represent the standard deviation.

## 3. Results

### 3.1. MICs and MBCs

As presented in Table 2, the MICs of TQ against *B. cereus* strains ranged from 4.0 to 8.0 μg/mL. TQ inhibited the growth of ATCC 14579, BR1, and BR4 at 4.0 μg/mL and exerted bactericidal effects at 8.0 μg/mL. The MIC and MBC of TQ were both found to be 8.0 μg/mL for CMCC 63301, CMCC 63303, M98, C7, and C58.

### 3.2. Growth Curve

**Figure 2:**
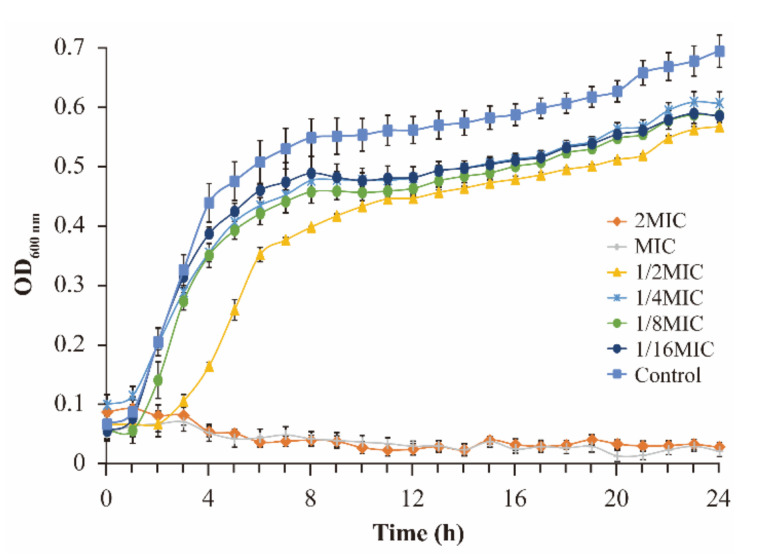
Growth curves of *Bacillus cereus* ATCC 14579 cultured in Luria–Bertani broth with various concentrations of thymoquinone (TQ).

The growth curve (Figure 2) indicated that TQ at MIC and 2 MIC completely inhibited the growth of *B. cereus* ATCC 14579 in LB broth after 24 h of incubation. The lag phase of *B. cereus* was lengthened, and the growth rate was lowered in the presence of 1/2 MIC TQ. In addition, in the presence of TQ at 1/16 MIC, 1/8 MIC, and 1/4 MIC, *B. cereus* displayed similar growth trends to the control, but these concentrations still slightly inhibited growth.

### 3.3. Inactivation of B. cereus by TQ in LB Broth

**Figure 3:**
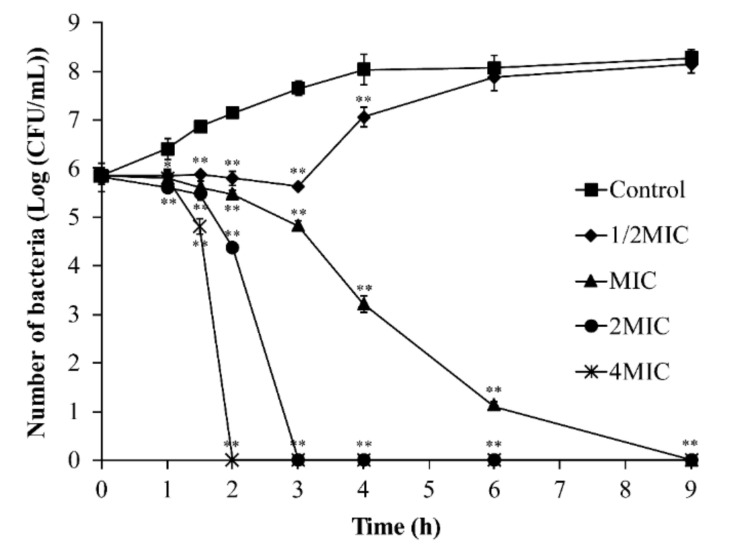
Inactivation of *Bacillus cereus* by thymoquinone (TQ) in Luria–Bertani broth. ** and * indicates statistical significance at *p* <0.01 and *p* < 0.05 compared with the control.

The bactericidal ability of TQ against *B. cereus* ATCC 14579 was tested at various concentrations from 0 (control) to 4 MIC after incubation at 37 °C for 9 h. As presented in Figure 3, the initial number of bacteria was similar among the treatment groups (approximately 5.8 log CFU/mL). The counts of *B. cereus* in the control group reached 8.0 log CFU/mL within 0–4 h and remained stable until 9 h. Whereas, at the 1/2 MIC concentration of TQ, *B. cereus* remained stable within 0–3 h and reached 7.9 log CFU/mL at 9 h. After *B. cereus* was treated with TQ at MIC for 6 h, the cell count was 1.1 log CFU/mL, and the count had decreased to below the detection limit (1 log CFU/mL) at 9 h. After TQ treatment at 2 MIC and 4 MIC for 3 and 2 h, respectively, the number of bacteria decreased to undetectable levels.

### 3.4. Antibacterial Mechanism of TQ against B. cereus

#### 3.4.1. Membrane Potential

**Figure 4:**
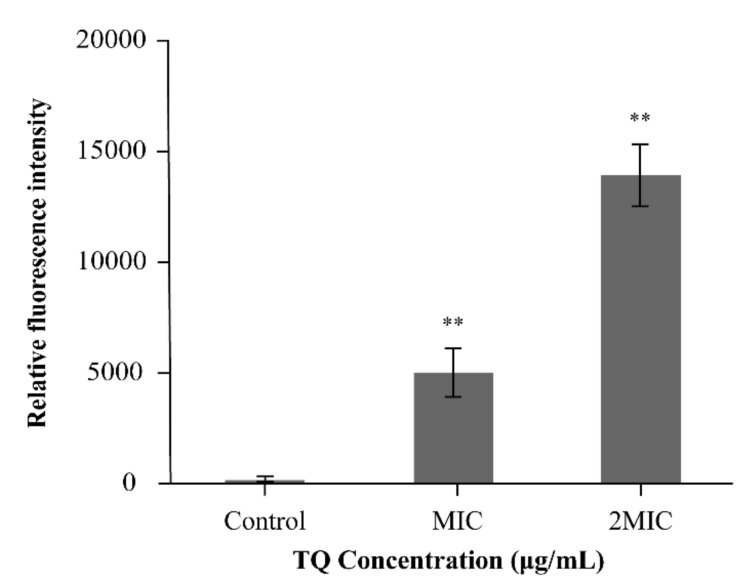
Effects of thymoquinone (TQ) on the membrane potential of *Bacillus cereus* ATCC 14579. ** indicates statistical significance at *p* < 0.01 compared with the control.

In comparison with the effects of the control, TQ induced rapid cell membrane depolarization in *B. cereus* ATCC 14579, as evidenced by the increase in fluorescence (positive values, Figure 4). Furthermore, the degree of depolarization deepened as the TQ concentration was increased from MIC to 2 MIC.

#### 3.4.2. Changes in Intracellular ATP Concentrations

**Figure 5:**
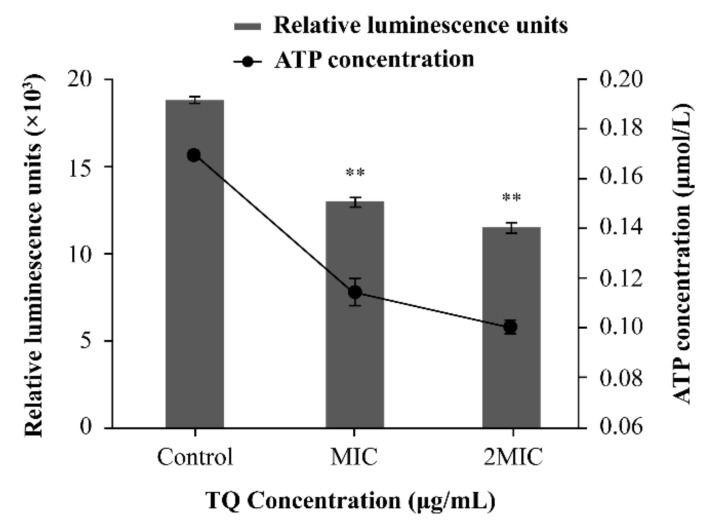
Effects of thymoquinone (TQ) on intracellular ATP concentrations in *Bacillus cereus* ATCC 14579. ** indicates statistical significance at *p* < 0.01 compared with the control.

There was good linearity between the relative luminescence units and ATP concentration (y = 100412x + 1500.2; R^2^ = 0.9997, data not displayed). As presented in Figure 5, compared with the effects of the control (0.17 ± 0.0037 μmol/L), the intracellular ATP concentrations of *B. cereus* decreased significantly (*p* < 0.01) following exposure to TQ at MIC (0.11 ± 0.0068 μmol/L) and 2 MIC (0.010 ± 0.0023 μmol/L).

#### 3.4.3. ROS Determination

**Figure 6:**
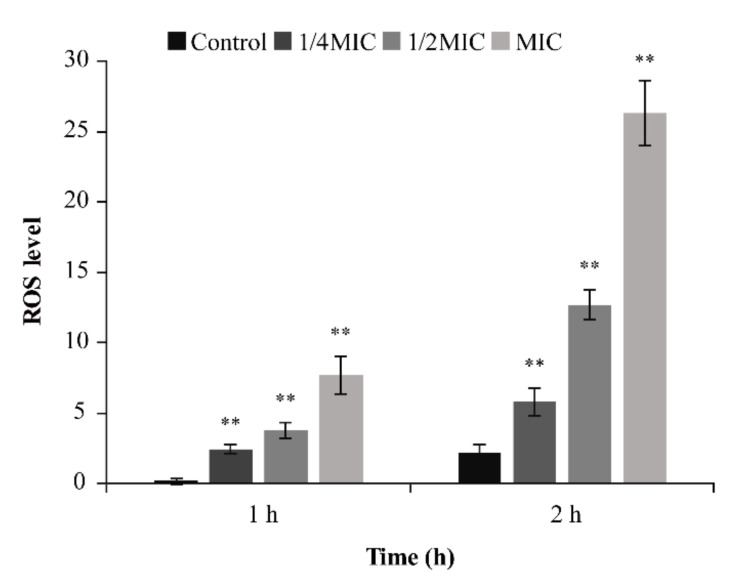
Effect of thymoquinone (TQ) treatment for 1 and 2 h on intracellular ROS levels in *Bacillus cereus*. ** indicates statistical significance at *p* < 0.01 compared with the control.

As illustrated in Figure 6, TQ significantly increased intracellular ROS levels in *B. cereus* ATCC 14579. When the treatment time was 1 h, 1/4 MIC TQ increased the ROS level by 9.60-fold relative to the control value, and 1/2 MIC and MIC TQ increased the ROS level by 14.37- and 31.08-fold, respectively. Similarly, when the treatment time was 2 h, the intracellular ROS level also increased significantly (*p <* 0.01), and ROS accumulation was obvious compared with that at 1 h.

#### 3.4.4. Morphology of *B. cereus*

**Figure 7:**
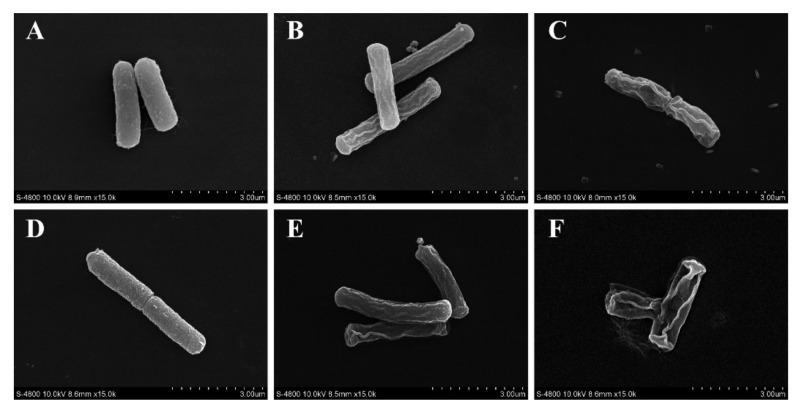
Field emission scanning electron microscopy observations (×15,000 magnification) of *Bacillus cereus* ATCC 14579. Untreated for 2 (**A**) and 4 h (**D**). Treated with thymoquinone (TQ) at 2 MIC for 2 (**B**) and 4 h (**E**). Treated with TQ at 4 MIC for 2 (**C**) and 4 h (**F**).

FESEM was performed to observe the morphological changes in *B. cereus.* Untreated *B. cereus* exhibited a typical rod-like structure with a smooth appearance (Figure 7A,D) Under the action of TQ at 2 MIC, the cell surface displayed undulating, rough, and wrinkled characteristics, and the degree of damage increased with increasing treatment time (2 and 4 h, Figure 7B,E). Moreover, cells treated with TQ at 4 MIC were severely shrunken, with notable depressions and deformity (Figure 7C,F). In addition, the extent of morphological damage increased with increasing treatment time.

#### 3.4.5. SDS-PAGE

**Figure 8:**
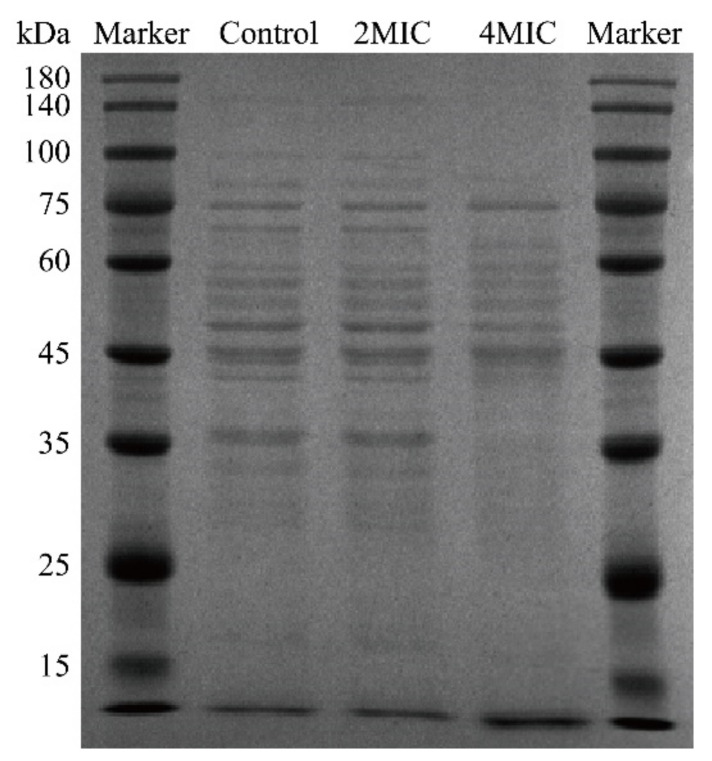
Sodium dodecyl sulfate–polyacrylamide gel electrophoresis analysis of intracellular soluble proteins from untreated and thymoquinone-treated *Bacillus cereus*.

The results of SDS-PAGE analysis of the soluble intracellular proteins of *B. cereus* are shown in Figure 8. The bands were clearer and more numerous in the control sample, and the bands of the samples obtained after TQ treatment at a concentration of 2 MIC displayed no obvious change compared with the control. However, as the concentration of TQ was raised to 4 MIC, some protein bands appeared with indistinct edges, became lighter in color (below 75 kDa), and were even lost (100–140 kDa). This suggests that TQ may have some effect on the cytosolic soluble protein of *B. cereus*, but the effect is not very prominent and shows only a weak effect at higher concentrations.

### 3.5. RT-qPCR Analysis

**Figure 9:**
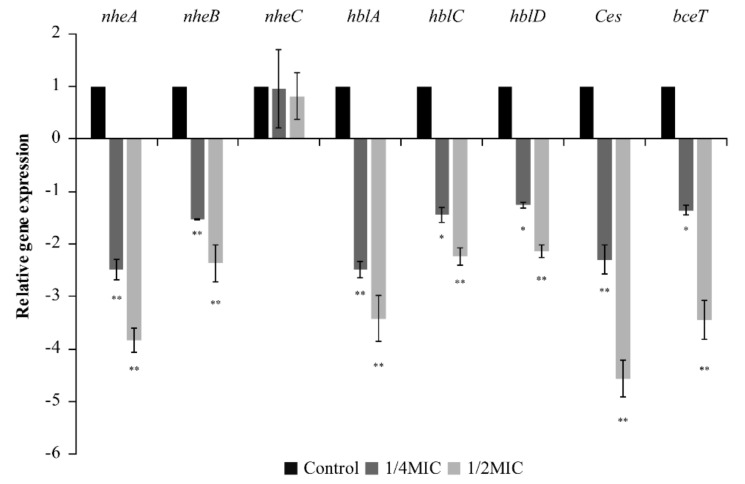
Effect of thymoquinone (TQ) on the transcription of *Bacillus cereus* ATCC 14579 toxin-related genes. ** and * indicates statistical significance at *p* < 0.01 and *p* < 0.05 compared with the control.

The effects of TQ on the transcript levels of toxin-related genes associated with *B. cereus* ATCC 14579 are presented in Figure 9. TQ at 1/4 MIC and 1/2 MIC decreased the transcription of genes related to non-hemolytic enterotoxins (*nheA*, *nheB*), hemolysins (*hblA*, *hblC*, *hblD*), vomitoxin (*Ces*), and enterotoxins (*bceT*) in *B. cereus*. The inhibitory effect of TQ was more pronounced at 1/2 MIC than at 1/4 MIC. However, TQ had no effect on non-hemolytic enterotoxin-related gene expression (*nheC*).

### 3.6. Determination of the Antibacterial Activity of TQ in RIF

**Figure 10:**
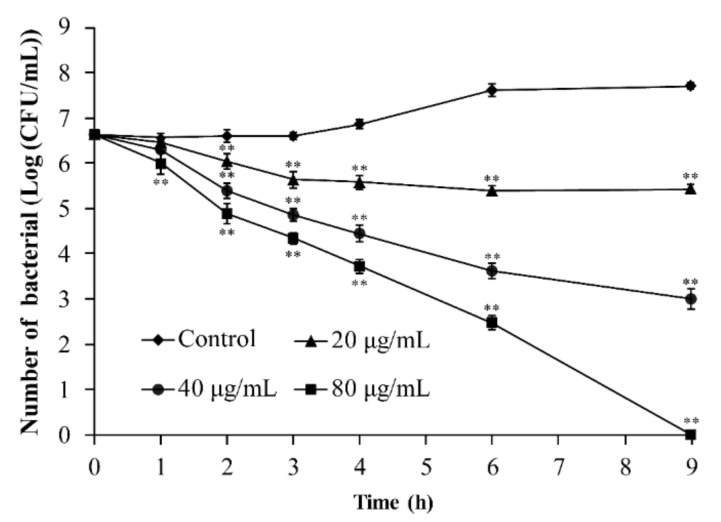
Analysis of reconstituted infant formula contaminated by *Bacillus cereus* inactivated by different concentrations of thymoquinone (TQ). ** indicates statistical significance at *p* < 0.01 compared with the control.

As presented in Figure 10, the initial count of *B. cereus* ATCC 14579 in RIF was approximately 6.63 log CFU/mL. At 25 °C, the number of cells in the control increased to approximately 7.60 log CFU/mL after 6 h and remained stable until 9 h. When the concentration of TQ was 20 and 40 μg/mL, the number of bacteria decreased by approximately 1.21 log CFU/mL and 3.62 log CFU/mL at 9 h, respectively. TQ at 80 μg/mL decreased the number of bacteria in RIF to undetectable levels (1 log CFU/mL) within 9 h.

### 3.7. Inhibitory Effect of TQ on B. cereus Spores

#### 3.7.1. Inhibition of Spore Germination by TQ

**Figure 11:**
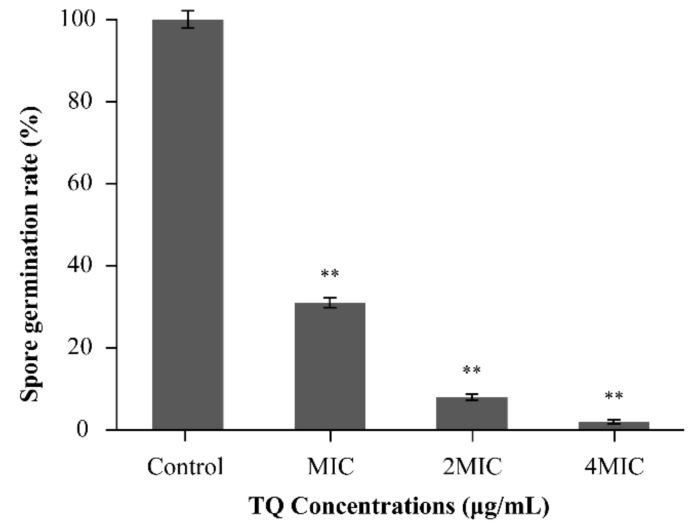
Spore germination rate following treatment with different concentrations of thymoquinone (TQ). ** indicates statistical significance at *p* < 0.01 compared with the control.

As presented in Figure 11, TQ had a significant (*p* < 0.01) inhibitory effect on the germination of *B. cereus* spores at concentrations ranging from MIC to 4 MIC. Compared with the effects of the control, the spore germination rate was only 30.95% after treatment with TQ at MIC. As the concentration of TQ increased to 2 MIC and 4 MIC, the spore germination rate decreased to 8.2% and 2.2%, respectively.

#### 3.7.2. CLSM Observations

**Figure 12:**
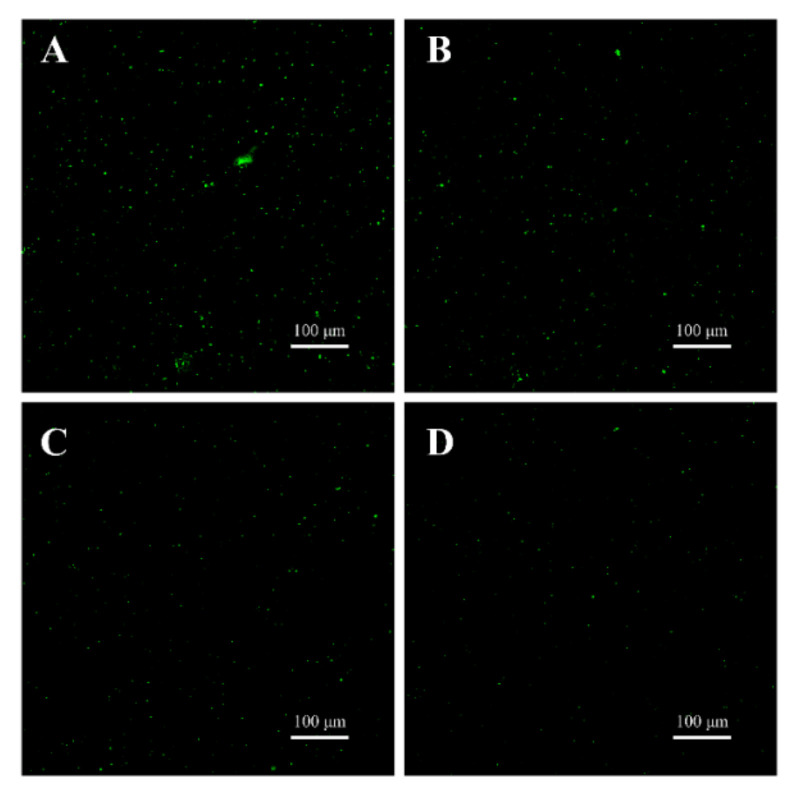
Confocal laser scanning microscopy observations (×200 magnification) of untreated *Bacillus cereus* spores (**A**) and spores treated with thymoquinone (TQ) at the minimum inhibitory concentration (MIC, **B**), 2 MIC (**C**), and 4 MIC (**D**).

The CLSM images of *B. cereus* spores are presented in Figure 12. The green fluorescence in the spores without TQ treatment was strong (Figure 12A). However, the green fluorescence was decreased by treatment with TQ at MIC (Figure 12B). Moreover, with increasing TQ concentrations, the green fluorescence in the field of view gradually weakened (Figure 12C,D).

## 4. Discussion

*B. cereus* is considered an important spoilage microorganism in the dairy industry, and it can contaminate dairy products environmentally [30]. Because of its ability to produce enterotoxins and vomitoxin, *B. cereus* can cause vomiting and diarrhea of sufficient severity to cause hospitalization and even death [31]. However, food contamination is often accompanied by spore production, and the commonly used methods to eliminate *B. cereus* such as pasteurization and ultraviolet radiation cannot effectively suppress spore germination. Consequently, treated food carries the risk of contamination caused by spore germination [28]. In previous studies, the antibacterial effect of natural extracts such as monolauroyl-galactosylglycerol and olive oil polyphenol extract on vegetative cells of *B. cereus* was reported [31,32], but the inhibitory effect on vegetative cells and spores of *B. cereus* has rarely been reported. Therefore, this study intended to evaluate the inhibitory effect of TQ on *B. cereus* from the aspects of vegetative cells and spores.

In this study, the MIC of TQ against *B. cereus* ranged from 4.0 to 8.0 μg/mL (Table 2). In previous reports, many natural active substances were found to have antibacterial activity against *B. cereus*. Kang et al. [33] reported that the MIC of thyme essential oil against *B. cereus* was 250 μg/mL. The MICs of monolauroyl-galactosylglycerol, mannosylerythritol lipids, and plantaricin JY22 against *B. cereus* were 156, 1250, and 6000 μg/mL, respectively [28,32,34]. Wang et al. [35] reported that thymol had antibacterial activity against *B. cereus*, with an MIC of 625 μg/mL. Among the natural substances reported in the above literature, TQ had the best antibacterial effect on *B. cereus*.

Membrane potential is an important parameter of microorganisms that reflects antibacterial material uptake and bactericidal action [36]. In this study, rapid depolarization of the cell membrane was detected in *B. cereus* after exposure to TQ (Figure 4). Previous studies found that depolarization also occurred in *B. cereus* after treatment with thyme essential oil [33]. In addition, Guo et al. [37] reported that MIC and 2 MIC *Amaranthus tricolor* crude extract could depolarize the cell membrane of *S. aureus*. Studies reported that membrane depolarization may be related to Na^+^ ion flow into cells following the opening of Na^+^ channels [38], and membrane depolarization is an important type of membrane damage [39]. Therefore, the change in membrane potential in *B. cereus* may be attributable to membrane damage caused by TQ, which results in Na^+^ ion inflow.

As an important parameter for evaluating the available energy of microorganisms, intracellular ATP plays an important role in many reactions across cell membranes, such as survival, metabolism, substance transport, and signal functions [40]. In this research, the intracellular ATP concentrations of *B. cereus* decreased significantly (*p <* 0.01) after exposure to TQ at MIC and 2 MIC for 30 min compared with the control (Figure 5). Similarly, Yang et al. [24] reported that coenzyme Q0 at concentrations of 0.2 and 0.4 mg/mL could decrease the intracellular ATP concentrations of *Salmonella typhimurium* to 21.9% and 4.7% of the control level, respectively. In addition, Shi et al. [41] found that lipoic acid could significantly reduce (*p <* 0.01) the intracellular ATP concentration in *Cronobacter sakazakii*. It has been reported that decreases in the intracellular ATP content in bacteria may be caused by changes in membrane permeability or rapid ATP hydrolysis [42]. The rapid hydrolysis of ATP may be required to restore the electrochemical gradient of cells [43].

ROS can cause various types of intracellular damage, including abnormal protein oxidation, DNA damage, and lipid peroxidation, thereby reducing cell viability [44]. In this study, TQ significantly increased the intracellular ROS level in *B. cereus* (Figure 6). In previous studies, Li et al. [45] found that 8, 16, and 32 μg/mL kalopanaxsaponin A increased intracellular ROS levels in *Candida albicans* by 13.48-, 19.08-, and 34.00-fold, respectively, of that in the control. Xiong et al. [46] demonstrated that the tea polyphenol epigallocatechin gallate could exert antibacterial effects by increasing the intracellular ROS level in *E. coli* op50. Mitochondria are both the power source of cells and the main source of ROS. When mitochondrial function is damaged, intracellular ROS levels are increased in bacteria, leading to further aggravation of mitochondrial damage [47]. ATP production decreases following mitochondrial damage, and increased ROS levels in bacteria also result in cell membrane damage [45], which is consistent with the results of ATP levels and membrane potential found in this study.

FESEM analysis revealed that TQ caused changes in the morphology of *B. cereus* (Figure 7). After exposure to TQ, the cell surface collapsed and contracted, and the cells’ morphology changed significantly. In previous reports, Shi et al. [48] found that 0.54 and 1.08 mg/mL citral could cause severe collapse of the surface of *C. sakazakii*. Sun et al. [49] confirmed by scanning electron microscopy that anthocyanins could damage the cell membrane of *L. monocytogenes*, *S. aureus*, *S. enteritidis*, and *Vibrio parahaemolyticus*. In addition, according to the results of ATP, membrane potential, and ROS experiments, the changes in *B. cereus* cell morphology may have been caused by TQ-induced membrane damage.

SDS-PAGE confirmed that TQ disrupted proteins or inhibited protein synthesis in *B. cereus* (Figure 8). Similarly, Wang et al. [50] found that coenzyme Q0 altered bacterial protein levels by damaging bacterial proteins or inhibiting their synthesis. Wang et al. [22] reported that lactic acid affects soluble proteins in *S. enteritidis*, *E. coli*, and *L. monocytogenes* by either disrupting bacterial proteins or inhibiting their synthesis. In this study, TQ decreased the intracellular ATP content of *B. cereus*, which may have led to insufficient energy for protein synthesis and resulted in reduced intracellular protein content.

*B. cereus* can produce a variety of toxins, including the protein complexes hemolysin BL (Hbl) and non-hemolytic enterotoxin (Nhe), the enterotoxin proteins enterotoxin t (BceT) and cytotoxin K (CytK), and vomitoxin [51]. In this study, TQ at 1/2 MIC and 1/4 MIC reduced the relative expression of the toxin production-related genes *nheA*, *nheB*, *nblA*, *nblC*, *nblD*, *Ces*, and *bceT* in *B. cereus* (Figure 9). The Hbl complex consists of three proteins transcribed from the genes *hblC*, *hblD*, and *hblA*. The Nhe complex is also composed of three different proteins encoded by the genes *nheA*, *nheB*, and *nheC*. The enterotoxins BceT and vomitoxin are encoded by the *becT* gene and the *Ces* gene, respectively [6]. The results indicated that TQ could effectively reduce the transcription of genes related to toxin production in *B. cereus*, thereby hindering the synthesis of hemolysin, non-hemolytic enterotoxin, enterotoxin, and cytotoxin K, and reducing the risk of food poisoning caused by *B. cereus*.

*B. cereus* is a common contaminant of milk and dairy products, and RIF contains high levels of nutrients such as proteins, vitamins, minerals, and water to support bacterial growth [3]. In the present study, TQ effectively inhibited *B. cereus* growth in RIF (Figure 10). Similarly, Bajpai et al. [52] reported that (−)-tetrahydroberberrubine acetate isolated from *Nandina domestica* extract reduced *B. cereus* contamination in rice. Voravuthikunchai et al. [53] found that rhodomyrtone isolated from *Rhodomyrtus tomentosa* could reduce the counts of *B. cereus* in pre-cooked rice and tuna steaks, but higher concentrations of antibacterial substances were needed for antibacterial effects in food, consistent with our results. The main reason is that compared with the laboratory medium used in in vitro studies, the availability of nutrients in a food matrix is higher, which may enable bacteria to repair damaged cells faster or resist environmental pressure. In addition to food ingredients, pH, water activity, and structural characteristics also affect the antibacterial activity of natural extracts [54]. In recent years, the technology of natural extract encapsulation has developed rapidly. Encapsulation technology can improve the thermal stability and chemical stability of natural extracts and improve enhance their solubility in solvents under certain conditions [55]. Therefore, entrapment may be one route for TQ’s application in the food industry.

In this study, TQ had an obvious inhibitory effect on spore germination in *B. cereus* (Figure 11). Similarly, mannosylerythritol lipids at 1.25 mg/mL reduced the spore germination rate of *B. cereus* to less than 2% [28]. To intuitively observe the effect of TQ on spore germination in *B. cereus*, CLSM was used. SYTO 16 is a membrane-permeable nucleic acid dye. It can only pass through the inner membrane after the spore completely germinates, and it exhibits strong green fluorescence upon binding to nucleic acid [56]. The experimental results demonstrated that the germination rate of spores decreased following TQ treatment (Figure 12), consistent with the results of the viable count method. Shu et al. [28] previously reported that mannosylerythritol lipids may inhibit spore germination by adsorbing onto the surface of *B. cereus* spores and destroying their proteins during spore germination. However, the mechanism by which TQ inhibited *B. cereus* spore germination requires further experimental investigation.

## 5. Conclusions

In summary, TQ has good antibacterial effects on *B. cereus*. TQ exerted its antimicrobial effects on *B. cereus* by causing depolarization of the cell membrane, decreasing intracellular ATP concentrations, increasing intracellular ROS levels, affecting intracellular protein synthesis, and altering the cellular morphology. In addition, TQ also purified RIF contaminated by *B. cereus*. RT-qPCR analysis confirmed that TQ downregulated the transcription of genes related to hemolysin, non-hemolytic enterotoxin, enterotoxin, and cytotoxin K production. Moreover, TQ significantly inhibited the germination of *B. cereus* spores. These findings suggest that TQ has a strong inhibitory effect on *B. cereus* and its spores, and has potential applicability in the food industry.

## Figures and Tables

**Table 1:** Reverse transcription–quantitative PCR primers used in this study for detecting different toxin-related genes.

Target Genes	Primers	Sequence of Primers (5′-3′)
16S rRNA	Forward	AGAGTTTGATCMTGGCTCAG
	Reverse	TACGGYTACCTTGTTACGACTT
*nheA*	Forward	AGGTAAATGCGATGAGTAG
	Reverse	TTGTTGAATGCGAAGAG
*nheB*	Forward	CAAGCTCCAGTTCATGCGG
	Reverse	GATCCCATTGTGTACCATTG
*nheC*	Forward	TGGATTCCAAGATGTAACG
	Reverse	ATTACGACTTCTGCTTGTGC
*Ces*	Forward	TTGTTGGAATTGTCGCAGAG
	Reverse	GTAAGCGAACCTGTCTGTAACAACA
*hblA*	Forward	GCTAATGTAGTTTCACCTGTAGCAAC
	Reverse	AATCATGCCACTGCGTGGACATATAA
*hblC*	Forward	AATCAAGAGCTGTCACGAAT
	Reverse	CACCAATTGACCATGCTAAT
*hblD*	Forward	AATGGTCATCGGAACTCTAT
	Reverse	CTCGCTGTTCTGCTGTTAAT
*bceT*	Forward	GACTACATTCACGATTACGCAGAA
	Reverse	CTATGCTGACGAGCTACATCCATA

**Table 2:** The MICs and MBCs of thymoquinone against *Bacillus cereus*.

Strains	Origin	MIC (μg/mL)	MBC (μg/mL)
ATCC 14579	ATCC	4.0	8.0
CMCC 63301	CMCC	8.0	8.0
CMCC 63303	CMCC	8.0	8.0
BR1	Rice	4.0	8.0
BR4	Rice	4.0	8.0
M98	Milk	8.0	8.0
C7	Milk	8.0	8.0
C58	Milk	8.0	8.0
